# Freshwater-Borne Bacteria Isolated from a Malaysian Rainforest Waterfall Exhibiting Quorum Sensing Properties

**DOI:** 10.3390/s140610527

**Published:** 2014-06-13

**Authors:** Wen-Si Tan, Nina Yusrina Muhamad Yunos, Pui-Wan Tan, Nur Izzati Mohamad, Tan-Guan-Sheng Adrian, Wai-Fong Yin, Kok-Gan Chan

**Affiliations:** Division of Genetics and Molecular Biology, Institute of Biological Sciences, Faculty of Science, University of Malaya, Kuala Lumpur 50603, Malaysia; E-Mails: tmarilyn36@gmail.com (W.-S.T.); ninayusrina@hotmail.com (N.Y.M.Y.); acelinetan38@yahoo.com (P.-W.T.); zetty_mohamad@yahoo.com (N.I.M.); adrian_tan_1991@yahoo.com (T.-G.-S.A.); yinwaifong@yahoo.com (W.-F.Y.)

**Keywords:** aquatic environment, *Chromobacterium violaceum* CV026, *Escherichia coli* [pSB401], *N*-acyl homoserine lactones (AHLs), quorum sensing

## Abstract

One obvious requirement for concerted action by a bacterial population is for an individual to be aware of and respond to the other individuals of the same species in order to form a response in unison. The term “quorum sensing” (QS) was coined to describe bacterial communication that is able to stimulate expression of a series of genes when the concentration of the signaling molecules has reached a threshold level. Here we report the isolation from aquatic environment of a bacterium that was later identified as *Enterobacter* sp.. *Chromobacterium violaceum* CV026 and *Escherichia coli* [pSB401] were used for preliminary screening of *N*-acyl homoserine lactone (AHL) production. The *Enterobacter* sp. isolated was shown to produce two types of AHLs as confirmed by analysis using high resolution tandem mass spectrometry. To the best of our knowledge, this is the first documentation of an *Enterobacter* sp. that produced both 3-oxo-C6-HSL and 3-oxo-C8-HSL as QS signaling molecules.

## 1. Introduction

Language plays a pivotal role in human communication while in bacterial cell-cell communication, it typically involves the production of small diffusible signaling molecules, a phenomenon known as quorum sensing (QS) [1]. QS is defined as the control of gene expression involving signaling molecules used by both Gram-negative and Gram-positive bacteria to regulate various physiological functions including secondary metabolite production, symbiosis and motility in response to cell density [2,3]. QS bacteria are present almost everywhere. including the human body and various environments. Gram-negative bacteria mostly produce signaling molecules belonging to the autoinducer-1 type, namely *N*-acyl homoserine lactones (AHLs) and Gram-positive bacteria use oligopeptide autoinducers [3,4]. To date, the only shared QS mechanism for both Gram-positive and Gram-negative bacteria involves the autoinducer-2 production by LuxS [3,4].

Intensive studies have been carried out, mainly focusing on AHLs [1–3,5,6]. The degree of saturation, fatty acid side chains that vary in chain length (ranging from 4–18 carbons) and the presence of hydroxyl-, oxo- or no substituents at the C3 position influence the characteristics of the AHL molecules [7]. Although they may have different lengths and degrees of saturation of the acyl side chain, the lactone ring structure is highly conserved [3,8]. The central mechanisms of AHL-driven QS are typically members of LuxI and LuxR proteins where AHL will bind to luxR and are activated as active transcriptional regulator proteins responsible for the regulation of gene activity [9]. The alteration of gene expression could lead to the activation of pathogenic factors such as intractable biofilm production, swarming and LasA protease formation [10,11].

The aquatic environment serves as a reservoir for microorganisms making it rich in bacterial physiological activities [12]. Microbial contamination of natural water is now becoming a major concern globally, particularly due to the presence of faecal material, agricultural or pasture runoff that often leads to increases in disease transmission to humans who utilize such water [13,14]. Furthermore, the presence of QS bacteria in the aquatic environment has attracted significant interest because QS regulates diverse bacterial functions including the expression of virulence factors [10,15]. A waterfall was chosen as the sampling source for bacterial isolation in this study. In Malaysia, there are many waterfalls that located inside tropical rainforests. In view of this, we investigated the presence of QS bacteria in a Malaysian tropical rainforest waterfall and we now report the isolation of a QS *Enterobacter* sp.

## 2. Experimental Section

### 2.1. Water Sampling and Isolation of Bacteria

Water sample collection was carried out in the year of 2013 at the top of the Sungai Tua Waterfalls which are located 10 km from Selayang and Ulu Yam. The GPS coordinates for this site were N 03 19.91′ E 101 42.15′. The water sample was collected at a depth of 12 cm below the water surface and kept in sterilized plastic bottles. The collected samples were kept at 4 °C until further analysis [16]. The water sample was serially diluted with saline buffer (0.9% NaCl) and spread onto Reasoner's 2A agar (in grams per litre: proteose peptone, 0.5; casamino acids, 0.5; yeast extract, 0.5; dextrose, 0.5, soluble starch, 0.5; dipotassium phosphate, 0.3; magnesium sulfate, 0.05; sodium pyruvate 0.3). Bacteria with observable different morphologies were isolated after incubation (24 h at 28 °C). Pure colonies was obtained with a few repeated cultures on Trypticase Soy (TS) medium (in grams per litre: tryptone, 10; soytone extract, 5; NaCl, 5; Bacto agar, 15).

### 2.2. Bacterial Strains, Culture Conditions and Biosensor Assay

The bacterial isolate M004 purified from the waterfall sample was selected for further work and routinely cultured on TS medium. *Chromobacterium violaceum*, CV026 served as AHL biosensor that will induce purple violacein pigmentation in response to the presence of AHLs with *N*-acyl side chains from C_4_ to C_8_ [17]. In addition, *Escherichia coli* [pSB401] was used as another *lux*-based biosensor that will produce bioluminescence in the presence of short chain AHLs [18]. For AHL screening, *Erwinia carotovora* GS101 and *E. carotovora* PNP22 served as positive and negative controls, respectively. All *C. violaceum* CV026, *E. coli* [pSB401], *E. carotovora* [GS101] and *E. carotovora* [PNP22] were routinely cultured on Lysogeny broth (LB) medium (in grams per litre: tryptone, 10; yeast extract, 5, NaCl, 5) and incubated at 28 °C. To solidify LB medium, 15 g/L Bacto agar was added.

### 2.3. Detection of AHLs Using C. violaceum CV026 and E. coli [pSB401] Biosensors

The isolate M004 was screened for AHL production by cross streaking the bacterial isolates close to the CV026 colony on a LB agar plate (24 h at 28 °C). Secondly, *E. coli* [pSB401] was also used as AHL biosensor to screen the production AHL. After 24 h incubation at 28 °C, a photon camera with 60 s of exposure was used to observe the induced bioluminescence [9].

### 2.4. Bacterial Strain Identification

Bacterial 16S rDNA genes were PCR-amplified with forward primer 27F [19] and reverse primer 1525R [20] using PCR mix (Promega Kit, Madison, WI, USA) while the genomic DNA was extracted using MasterPure™ DNA Purification Kit (Epicentre Inc., Madison, WI, USA). PCR amplification and purification was carried out as described previously [7]. PCR product sequence alignment was done using GenBank BLASTN program followed by phylogenetic analysis using the Molecular Evolutionary Genetics Analysis (MEGA) version 6.0 [21,22].

### 2.5. Extraction of AHLs from Bacteria Culture

Bacteria (with positive AHL production) were cultured in LB broth buffered to pH 5.5 with 50 mM of 3-(*N*-morpholino)propanesulfonic acid (MOPS) in an incubator shaker (200 rpm; 28 °C; 18 h) [8]. The spent supernatant was extracted twice with equal volume of acidified (0.1% *v*/*v* glacial acetic acid) ethyl acetate as described previously [23]. The organic solvent was dried in fume hood and the dried extracts were resuspended in 1 mL of acidified ethyl acetate and completely dried. Finally, 200 μL of acetonitrile (HPLC grade) was added and the mixture vortexed to dissolve the extracts. The mixture was then centrifuged at 12,000 rpm for 5 min to remove any insoluble residue. The dissolved sample (an aliquot of 75 μL) was withdrawn and placed in sample vials for mass spectrometry analysis.

### 2.6. AHL Profiling by Mass Spectrometry (MS)

The analysis of AHL by MS was carried out as described previously [23]. The flow rate and mobile phases were as reported [24]. The high-resolution electro-spray ionization mass spectrometry (ESI-MS) was performed with an Agilent 6500 Q-TOF LC/MS system (Agilent Inc., California, CA, USA) and was carried out in the ESI-positive mode [23,24]. The precursor ion target of *m*/*z* 102 indicates the [M+H]^+^ ion of the core lactone ring moiety, the *m*/*z* value range detection, and the mass spectra data analysis were performed as reported [24].

### 2.7. Biofilm Assay

The biofilm assay was done as described previously [25,26] with slight modifications. The overnight culture of strain M004 was diluted with LB medium and adjusted to OD_600_ of 0.1. Next, 50 μL of the diluted culture was added to 930 μL of LB medium supplemented with 1, 2, and 3 mg/mL of anti-QS compounds (catechin [25] and malabaricone C [26]) in a microtitre plate. The M004 cultures were treated with and without DMSO and served as negative and positive controls, respectively. The M004 cells with different culture conditions were incubated statically for 72 h at 28 °C. The planktonic bacteria were removed by washing thrice with sterile distilled water [27] and the plate was air-dried for 15 min and was stained with 200 μL of 0.1% (*w*/*v*) crystal violet per well for 30 min. After staining, the excess crystal violet was removed and washed with sterile distilled water twice. The quantitative analysis of biofilm production was done by adding 200 μL of 95% (*v*/*v*) ethanol and 100 μL of resulting solution was transferred to a new microtitre plate. The absorbance of the solution was read at OD_590_ with microplate reader. All experiments were performed in triplicate.

## 3. Results and Discussion

### 3.1. Isolation and Screening of AHL Producing Bacteria

The study aimed to detect AHL-producing bacteria from a tropical rainforest waterfall. The temperature of the water was 25 °C during the daytime and the pH value was 7 when sampling was done. The water samples were collected at the most top of the waterfall with less human activities to reduce the faecal contamination during water collection [28,29].

There are many types of bacterial biosensors available for screening of AHL production [9,17,18]. These biosensors carried a defective AHL synthase but contain a functional LuxR-family protein cloned together with a cognate target promoter which is able to positively regulate the reporter gene such as violacein pigment production and bioluminescence induction [9]. In this study, CV026 was employed for preliminary screening due to the rapidness and accuracy that it can provide [17]. Subsequently, *E. coli* [pSB401] was also utilized as second biosensors for preliminary screening.

**Figure 1.**
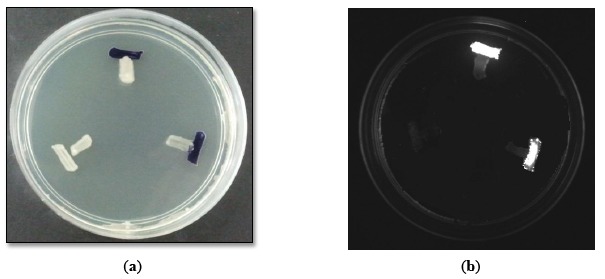
(**a**) AHL screening of strain M004 with CV026. *E. carotovora* PNP22 and *E. carotovora* GS101 served as negative and positive controls respectively. (**b**) AHL screening of strain M004 with *E. coli* [pSB401]. *E. carotovora* PNP22 and *E. carotovora* GS101 served as negative and positive controls, respectively.

Preliminary screening of the AHL production with CV026 (Figure 1a) and *E. coli* [pSB401] (Figure 1b) indicated that the M004 strain produced short chain AHLs [17,18]. This strain was then subjected to molecular identification.

### 3.2. Molecular Identification of Bacterial Strain

**Figure 2.**
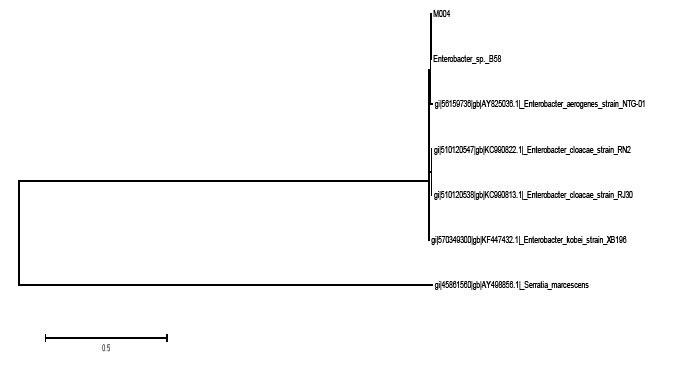
The evolutionary history was inferred by using the Maximum Likelihood method based on the Tamura-Nei model.

The identity of strain M004 was confirmed by 16S rDNA gene sequencing showing that it clustered within the *Enterobacter* genus. The strain shared 99% similarity in the BLAST search. Based on the phylogenetic tree constructed (Figure 2), strain M004 may represents a new species of the *Enterobacter* genus. More analysis should be performed to further confirm this finding.

The tree with the highest log likelihood (−2236.6968) is shown in Figure 2. Initial tree(s) for the heuristic search were obtained automatically as reported elsewhere [30]. There were a total of 1440 positions in the final dataset. Evolutionary analyses were conducted in MEGA6.

### 3.3. Identification of AHL Production

There are several documented *Enterobacter* infections which include bacteremia, lower respiratory tract infections, soft tissue infections and osteomyelitis [31–33]. They are known as opportunistic pathogens and its virulence seems to be due largely to an endotoxin. Environmental reservoirs containing opportunistic pathogens are a source of some public health concern [32]. To date, there are several reports that document the isolation of *Enterobacter* from environmental samples. Esiobu and co-workers isolated antibiotic-resistant *Enterobacter* sp. from fresh water [32]. Thus, the availability to detect AHL profile of *Enterobacter* sp. represents the key step to understand the QS-dependent virulence of this potential opportunistic pathogen.

**Figure 3.**
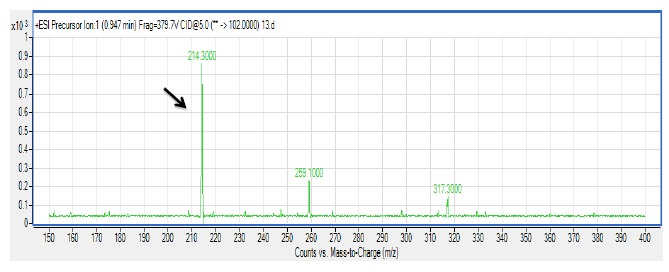

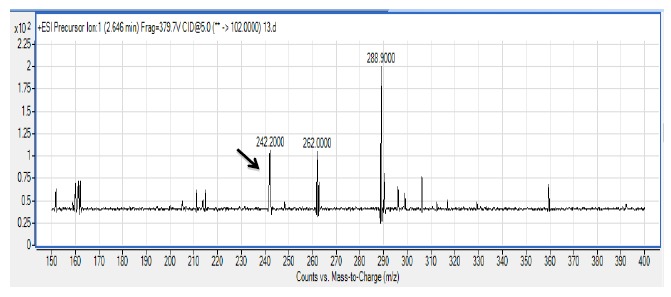
Mass spectrometry analysis of spent supernatants extract *Enterobacter* sp. strain M004. (**Upper Panel**)**:** mass spectra of 3-oxo-C6 HSL (*m*/*z* 214.3000) (marked by arrow). (**Lower Panel**)**:** mass spectra of 3-oxo-C8 HSL (*m*/*z* 242.2000) (marked by arrow).

There are several members in the *Enterobacter* genera displaying QS properties such as *Enterobacter sakazakii* that are considered as an opportunistic pathogen that is often associated with food-borne diseases such as meningitis or enteritis [33]. As reported by Angelika and colleagues, *E. sakazakii* produces 3-oxo-C6-HSL and 3-oxo-C8-HSL, indicating its pathogenicity is achieved via cell-to-cell signaling molecules [33]. Other work done by Yin and colleagues further showed that a member of the *Enterobacter* genus exhibits long chain AHL production, namely of *N*-dodecanoyl-l-homoserine lactone (C12-HSL) [34]. The spent culture supernatant of M004 strain was analyzed using mass spectrometry analysis which confirmed the presence of 3-oxo-C6-HSL and 3-oxo-C8-HSL (Figure 3). To our best knowledge, this is the first documentation of *Enterobacter* sp. strain M004 isolated from a waterfall and its production of both 3-oxo-C6-HSL and 3-oxo-C8-HSL. Hence, our group is currently expanding this work to the whole genome sequence in order to gain insights on the QS luxI/R system in this strain.

### 3.4. Biofilm Formation of Enterobacter sp. Strain M004

**Figure 4.**
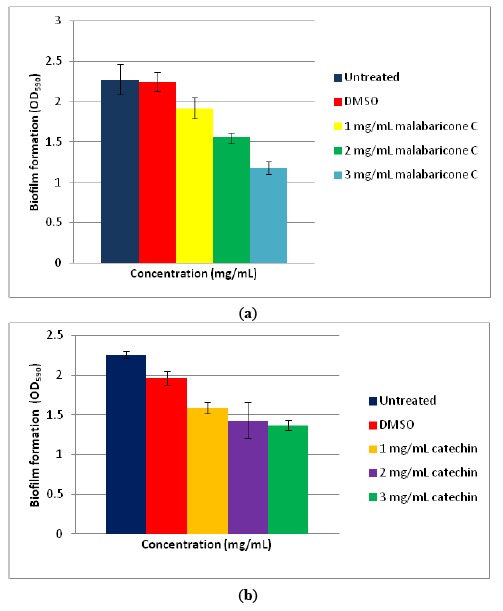
(**a**) Qualitative analyses of violacein inhibition by anti-QS compound, malabaricone C and (**b**) catechin with three different concentrations. Bars: standard errors of the mean.

Biofilm formation is often a QS-regulated phenotype [26,27]. The formation of biofilms often initiates with bacteria colonization followed by surface attachment and finally biofilm development and maturation. The ability of bacteria to form biofilms is often linked to pathogenic traits during chronic infection. There are several reports that the members of *Enterobacter* possess the ability to form biofilms [27,33]. *Enterobacter* sp. M004 has been shown to be able to form biofilms (Figure 4). In our study, we used two anti-QS compounds that have been reported, namely catechin [25] and malabaricone C [26], to inhibit the biofilm formation by *Enterobacter* sp., strain M004 whereby both catechin and malabaricone C reduced the biofilm formation in *Enterobacter* sp., strain M004 in a dose-dependent manner (Figures 4).

Since QS regulates a battery of bacterial virulence factors [15] hence this work illustrated the significance in expanding the research on AHL-producing bacteria present in environmental samples. Isolation of QS bacteria from fresh water may indicate potential virulence of this isolate, so this work also suggests that fresh water may be a potential reservoir for QS pathogens that should be given appropriate attention. We are currently conducting whole genome sequencing on *Enterobacter* sp. strain M004 aiming to isolate the AHL synthase and receptor genes that will provide more insight into the QS regulatory system in this bacterium.

## 4. Conclusions/Outlook

We report here the AHL profile of *Enterobacter* sp. M004 isolated from an environmental water sample. Two AHLs, namely 3-oxo-C6 HSL and 3-oxo-C8 HSL, were detected in the spent culture supernatant of isolate M004. *Enterobacter* sp. M004 has been shown to be able to form biofilms which could be inhibited by anti-QS compounds, suggesting that it is QS-dependent trait. To the best of our knowledge, this is the first documentation of this *Enterobacter* sp. that produces both 3-oxo-C6 HSL and 3-oxo-C8 HSL.
